# Supplementary material for: Phenotype-driven precision oncology as a guide for clinical decisions one patient at a time
Source: Nat Commun. 2017 Sep 5;8:435. doi: 10.1038/s41467-017-00451-5 (PMC5585361; doi:10.1038/s41467-017-00451-5)
Supplement: Supplementary file 1 — Supplementary Information [file 41467_2017_451_MOESM1_ESM.pdf]

Description of Supplementary Files

File Name: Supplementary Information
Description: Supplementary Figures and Supplementary Tables

File Name: Peer Review File

a

STR profile marker analysis

Marker	HN137 Blood	HN137 Pri Tumour	HN137 Met Tumour	HN137 Pri PDX	HN137 Met PDX	HN137 Pri Cell line	HN137 Met Cell line
AMEL	X, Y	X, Y	X, Y	X	X	X	X
CSF1PO	10	10	10	10	10	10	10
D13S317	8, 9	8, 9	8, 9	8	8	8	8
D16S539	9, 11	9, 11	9, 11	9, 11	9, 11	9, 11	9, 11
D18S51	14, 15	14, 15	14, 15	14, 15	14, 15	14, 15	14, 15
D21S11	29, 30	29, 30	29, 30	29, 30	29	29, 30	29
D3S1358	15, 16	15, 16	15, 16	16	16	16	16
D5S818	11, 14	11, 14	11, 14	14	14	14	14
D7S820	9, 11	9, 11	9, 11	9, 11	9, 11	9, 11	9, 11
D8S1179	12, 13	12, 13	12, 13	12, 13	12, 13	12, 13	12, 13
FGA	22, 23	22, 23	22, 23	22, 23	22, 23	22, 23	22, 23
Penta D	8, 9	8, 9	8, 9	8, 9	9	8, 9	9
Penta E	11, 12	11, 12	11, 12	11, 12	11, 12	11, 12	11, 12
TH01	7, 9	7, 9	7, 9	7, 9	7, 9	7, 9	7, 9
TPOX	8, 11	8, 11	8, 11	8, 11	8, 11	8, 11	8, 11
vWA	17, 19	17, 19	17, 19	17	17	17	17

b

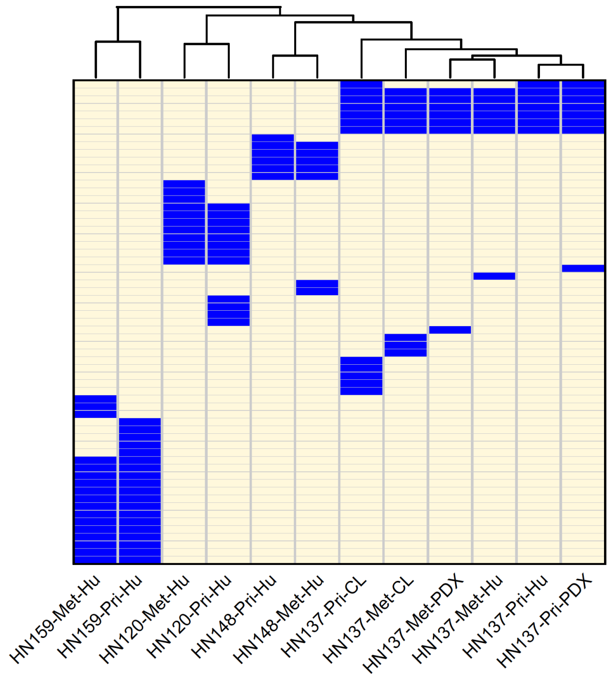

c

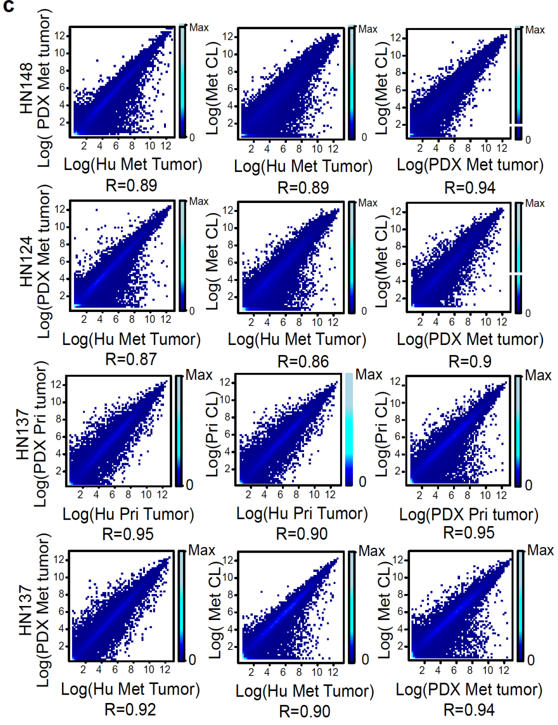

d

e

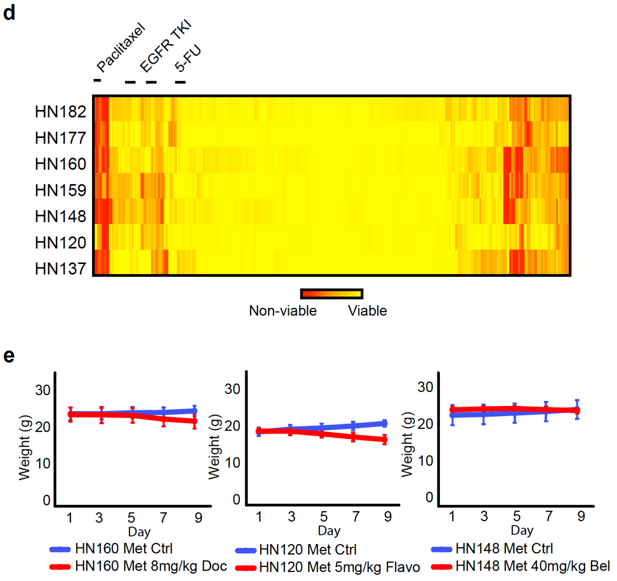

Supplementary Figure 1

(**a**) Analysis of various HN137-derived models' genetic profile based on 16 STR markers. (**b**) Genomic comparison of single nucleotide variants (SNV) across 5 pairs of (primary and metastatic) patient tumors, and across various HN137 models (Patient tumor (Hu), PDX, and Cell line (CL)). Blue denotes presence (allelic frequency > 0.10), while white denotes absence of SNP (**c**) Pair-wise gene expression correlations across HN148, HN124 and HN137 tumor (Hu Tumor), PDX tumor (PDX Tumor) and PDC cell line (CL) models for both primary and/or metastatic tumors. (**d**) Unsupervised clustering of heatmap that shows response of various PDC lines tested against the SelleckChem anti-cancer compound library at 1 μM in triplicates. The viabilities of cells in the presence of 317 small molecules inhibitors are compared across all patients. Several standard-of-care and promising targeted treatments e.g. epidermal growth factor receptor tyrosine kinase inhibitors (EGFR-TKI), 5-Fluorouracil (5'FU), and Paclitaxel are highlighted. (**e**) Body weight of male mice, bearing HN160-Met PDX (left panel), HN120-Met PDX (center panel) and HN148-Met PDX (right panel) receiving control treatment (Ctrl), 8 mg kg^{-1} Docetaxol (Doc), 5 mg kg^{-1} Flavopiridol (Flavo) and 40 mg kg^{-1} Belinostat (Bel) respectively.

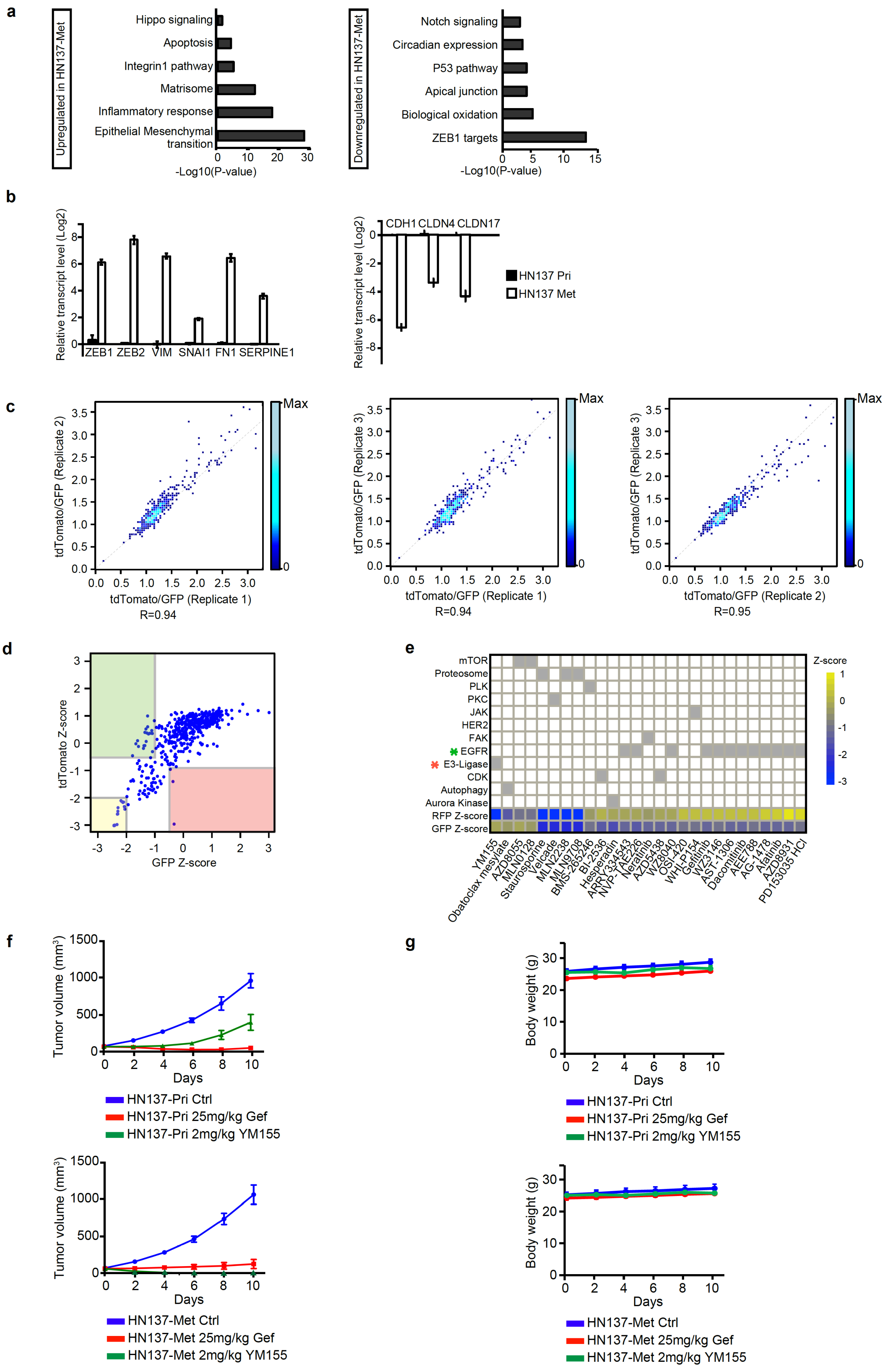
a
Upregulated in HN137-Met
Hippo signaling
Apoptosis
Integrin1 pathway
Matrisome
Inflammatory response
Epithelial Mesenchymal transition
-Log10(P-value)
Downregulated in HN137-Met
Notch signaling
Circadian expression
P53 pathway
Apical junction
Biological oxidation
ZEB1 targets
-Log10(P-value)
b
Relative transcript level (Log2)
ZEB1 ZEB2 VIM SNAI1 FN1 SERPINE1
CDH1 CLDN4 CLDN17
HN137 Pri
HN137 Met
c
tdTomato/GFP (Replicate 2)
tdTomato/GFP (Replicate 1)
R=0.94
tdTomato/GFP (Replicate 3)
tdTomato/GFP (Replicate 1)
R=0.94
tdTomato/GFP (Replicate 3)
tdTomato/GFP (Replicate 2)
R=0.95
Max
d
tdTomato Z-score
GFP Z-score
e
mTOR
Proteosome
PLK
PKC
JAK
HER2
FAK
EGFR
E3-Ligase
CDK
Autophagy
Aurora Kinase
RFP Z-score
GFP Z-score
Z-score
YM155
Obatoclax mesylate
AZD8055
MLN0128
Staurosporine
Velcade
MLN2238
MLN9708
BMS-265246
BI-2536
Hesperadin
ARRY334543
NVP-TAE226
Neratinib
AZD5438
WZ8040
OSI-420
WHI-P154
Gefitinib
WZ3146
AST-1306
Dacomitinib
AEE788
AG-1478
Afatinib
AZD8931
PD153035 HCl
f
Tumor volume (mm³)
Days
HN137-Pri Ctrl
HN137-Pri 25mg/kg Gef
HN137-Pri 2mg/kg YM155
HN137-Met Ctrl
HN137-Met 25mg/kg Gef
HN137-Met 2mg/kg YM155
g
Body weight (g)
Days
HN137-Pri Ctrl
HN137-Pri 25mg/kg Gef
HN137-Pri 2mg/kg YM155
HN137-Met Ctrl
HN137-Met 25mg/kg Gef
HN137-Met 2mg/kg YM155

Supplementary Figure 2

(**a**) Molecular signatures ($P < 0.05$) of genes that are upregulated (left panel) and downregulated (right panel) in HN137-Met compared to HN137-Pri cells. (**b**) qPCR validation of mesenchymal marker genes that are upregulated (left panel) and epithelial markers that are downregulated (right panel) in HN137-Met in comparison to HN137-Pri. Triplicate data, each normalized against *GAPDH*, are represented as mean ± s.d. (**c**) Scatterplot of correlation between screen replicates ($n = 3$). (**d**) Scatterplot of the distribution of the RFP and GFP Z-scores for each compound. Compounds in the red region are HN137-Met-specific (RFP Z < -1; GFP Z > -0.5), while compounds in the green region are HN137-Pri-specific (GFP Z < -1; RFP Z > -0.5). Compounds in the yellow region indicate dual cytotoxicity (GFP Z < -2, RFP Z < -2). (**e**) Heatmap of Z-scores and putative targets of selected hit compounds. Green astericks highlights EGFR class of compounds showing selectivity for Primary while red astericks highlights YM155 that show selectivity for Met. (**f**) 6 independent cohorts of mice ($n = 5$), engrafted with HN137-Pri PDX (top panel) or HN137-Met PDX (bottom panel) on both flanks were treated with vehicle control (Ctrl), 25 mg kg^{-1} Gefitinib (Gef) and 2 mg kg^{-1} YM155. (**g**) Body weights of male mice, bearing HN137-Pri and HN137-Met PDX receiving control treatment (Ctrl), 25 mg kg^{-1} of Gefitinib (Gef) and 2 mg kg^{-1} of YM155.

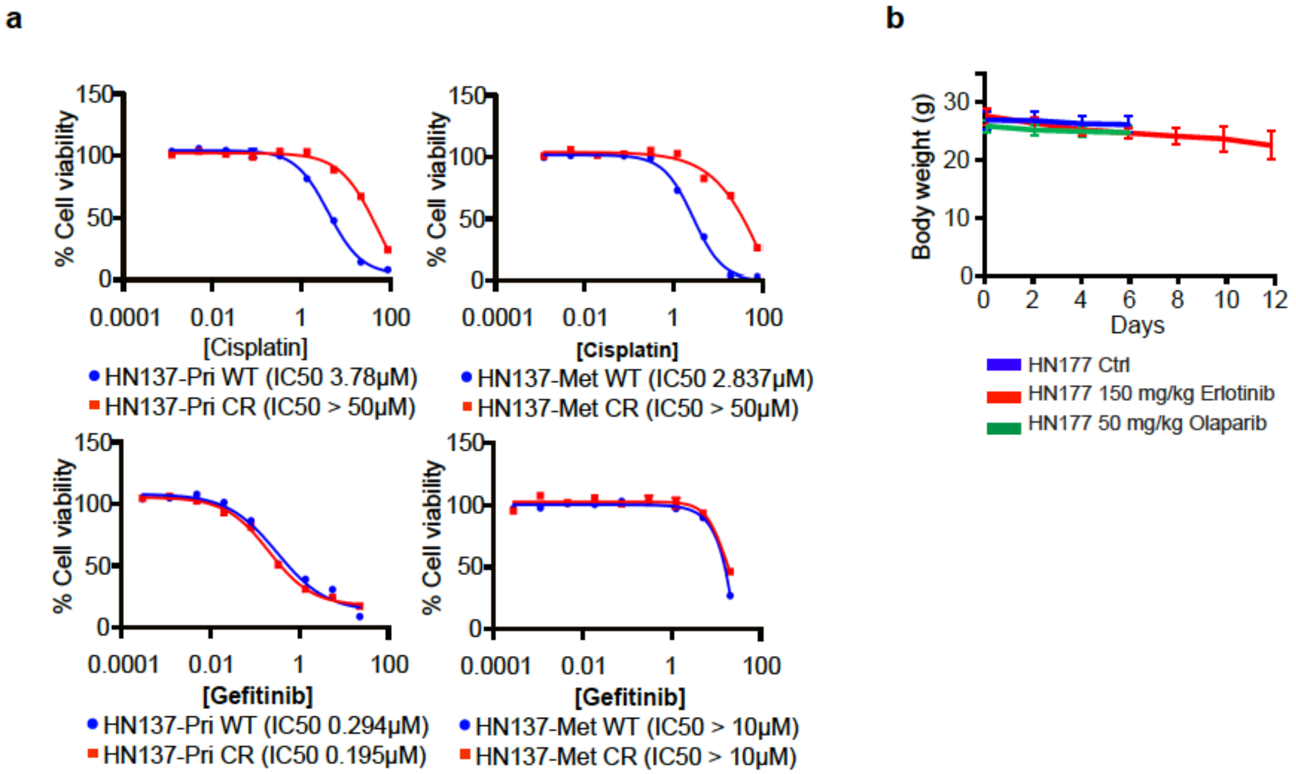

Supplementary Figure 3

(**a**) HN137-Pri, HN137-Met and corresponding Cisplatin-resistant (CR) cells were tested for Cisplatin (upper panel) and Gefitinib (lower panel) sensitivity. Experiments were performed at least twice, in triplicates. Cell viability was determined using CellTiter-Glo reagent. Error bars represent mean ± s.d. (**b**) Body weights of male mice, bearing HN177-PDX receiving vehicle control (Ctrl), 150 mg kg^{-1} Erlotinib and 50 mg kg^{-1} Olaparib.

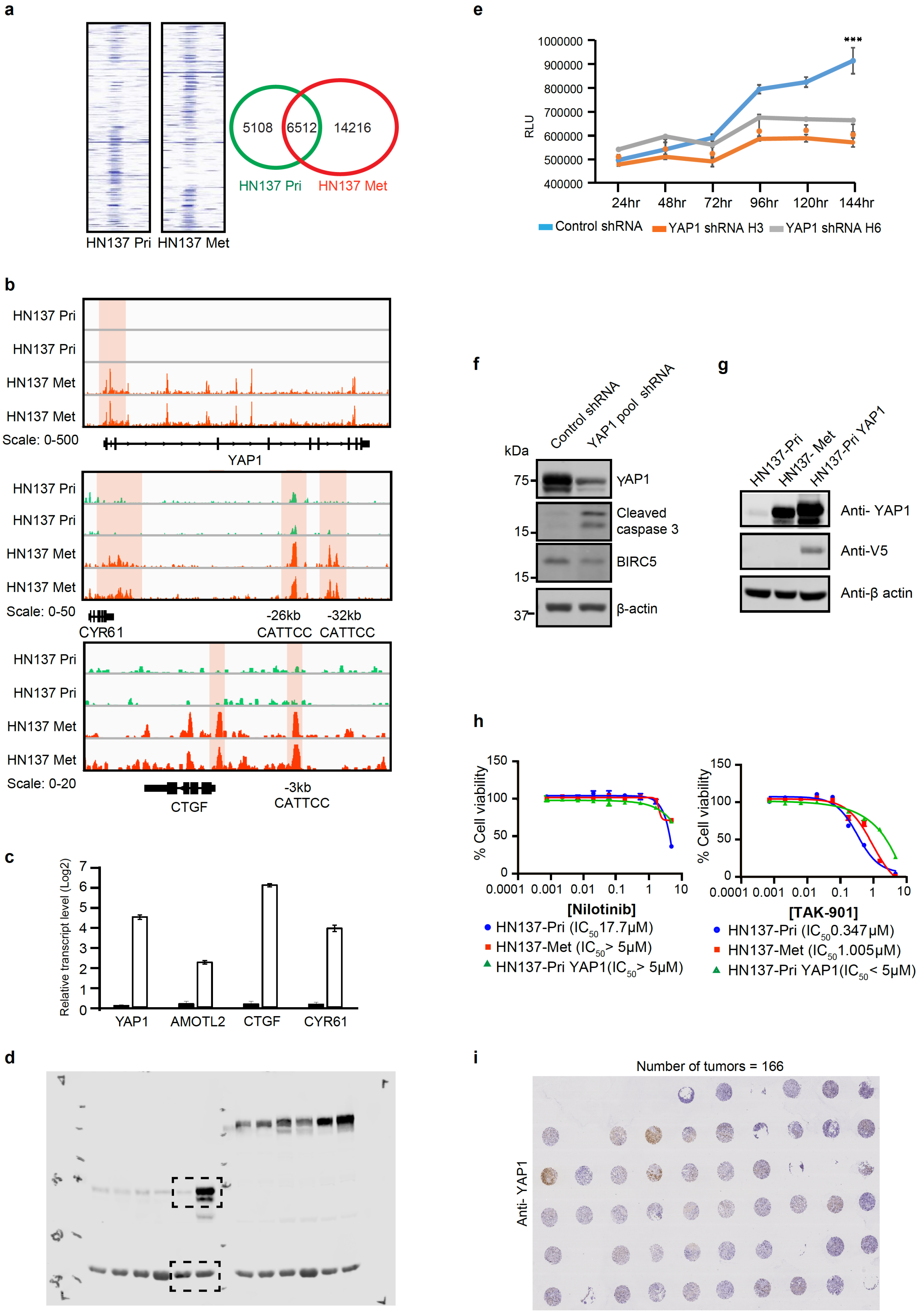
a
HN137 Pri
HN137 Met
5108
6512
14216
HN137 Pri
HN137 Met
b
HN137 Pri
HN137 Pri
HN137 Met
HN137 Met
Scale: 0-500
YAP1
Scale: 0-50
CYR61
-26kb CATTCC
-32kb CATTCC
Scale: 0-20
CTGF
-3kb CATTCC
c
Relative transcript level (Log2)
YAP1
AMOTL2
CTGF
CYR61
d
e
RLU
***
24hr
48hr
72hr
96hr
120hr
144hr
Control shRNA
YAP1 shRNA H3
YAP1 shRNA H6
f
kDa
Control shRNA
YAP1 pool shRNA
YAP1
Cleaved caspase 3
BIRC5
β-actin
g
HN137-Pri
HN137- Met
HN137-Pri YAP1
Anti- YAP1
Anti-V5
Anti-β actin
h
% Cell viability
[Nilotinib]
HN137-Pri (IC50 17.7μM)
HN137-Met (IC50 > 5μM)
HN137-Pri YAP1(IC50 > 5μM)
[TAK-901]
HN137-Pri (IC50 0.347μM)
HN137-Met (IC50 1.005μM)
HN137-Pri YAP1(IC50 < 5μM)
i
Number of tumors = 166
Anti- YAP1

Supplementary Figure 4

(**a**) Global open chromatin (FAIRE-seq) profiles of HN137-Pri (left) and HN137-Met (right) cells. Venn diagram depicting number of open chromatin sites in HN137-Pri (green circle) and HN137-Met (red circle) cells. (**b**) Open chromatin regions were detected in promoter and enhancer regions of *YAP1* and its target genes. FAIRE-seq peaks depicted were found to be statistically significant (FDR < 10^{-4}). (**c**) qPCR of *YAP1* and its target genes (right panel). Triplicate data, each normalized against *GAPDH*, are represented as mean ± s.d. (**d**) Uncropped western blot for Fig 4c. (**e**) 5-day growth curves of HN137-Met cells transiently transfected with Control (CT) shRNA and *YAP1* shRNA. *YAP1* shRNA H3 and *YAP1* shRNA H6 represent different *YAP1* shRNA sequences. Triplicate data, error bars represent mean ± s.d. Two-tail Student's t-tests comparing viability between control and *YAP1* shRNA treated cells was found to be statistically significant, ***P < 0.001. (**f**) Western blot analysis of YAP1, cleaved-caspase 3, and BIRC5 upon YAP1 downregulation in HN137-Met cells. (**g**) Western blot for YAP1 expression in HN137-Pri, HN137-Met and HN137-Pri stably overexpressing YAP1 (HN137-Pri YAP1). (**h**) Dose response of HN137-Pri, HN137-Met, and HN137-Pri YAP1 to Nilotinib (left panel) and TAK-901 (right panel). Experiments were performed at least twice, in triplicates. Cell viability was determined using CellTiter-Glo reagent. Error bars represent mean ± s.d. (**i**) Representative images of immunohistochemistry staining of oral squamous cell carcinoma tissue microarray stained using YAP1 antibody.

Supplementary Table 1
List of mutations across various patient's tumor and models

Sample	Chr	Position	Reference	Consensus	Allele Frequency	Gene	Change Type	AA change
HN159-Pri-Hu	chr17	7577124	C	M	0.246588	TP53	Nonsyn	V272L
HN159-Pri-Hu	chr14	75022279	G	R	0.2822515	LTBP2	Syn_or_UTR	-
HN159-Pri-Hu	chrX	102754975	C	Y	0.2583145	RAB40A	Nonsyn	R237Q
HN159-Pri-Hu	chr11	118347634	C	Y	0.2084945	MLL	Nonsyn	P1091S
HN159-Pri-Hu	chr17	76993389	G	R	0.183217	CANT1	Nonsyn	R106*
HN159-Pri-Hu	chr3	121206503	G	K	0.1696535	POLQ	Nonsyn	P1759T
HN159-Pri-Hu	chr2	80846308	T	Y	0.1682035	CTNNA2	Syn_or_UTR	-
HN159-Pri-Hu	chr2	80085319	A	R	0.1513745	CTNNA2	Intron_or_UTR	-
HN159-Pri-Hu	chr6	152841563	C	Y	0.14265	SYNE1	Intron_or_UTR	-
HN159-Pri-Hu	chr10	90771790	G	S	0.1412815	FAS	Nonsyn	K201N
HN159-Pri-Hu	chr13	20600757	T	Y	0.1335805	ZMYM2	Syn_or_UTR	-
HN159-Pri-Hu	chr9	87375171	C	Y	0.118797	NTRK2	Intron_or_UTR	-
HN159-Pri-Hu	chr15	66777347	C	S	0.1160375	MAP2K1	Nonsyn	T238S
HN159-Pri-Hu	chr8	31004677	G	S	0.1043465	WRN	Intron_or_UTR	-
HN159-Pri-Hu	chr9	87374760	C	S	0.1075255	NTRK2	Intron_or_UTR	-
HN159-Pri-Hu	chr20	57428917	C	M	0.1056875	GNAS	Nonsyn	P136Q
HN137-Met-cell-line	chr1	47735383	C	M	0.203365	STIL	Nonsyn	A847S
HN137-Met-cell-line	chr1	65332664	A	W	0.556698	JAK1	Nonsyn	L292Q
HN137-Met-cell-line	chr10	70332852	C	Y	0.2984785	TET1	Nonsyn	P253S
HN137-Met-cell-line	chr12	40677649	A	W	0.280722	LRRK2	Intron_or_UTR	-
HN137-Met-cell-line	chr17	7578176	C	T	0.9990775	TP53	Nonsyn	V186I
HN137-Met-cell-line	chrX	53226022	G	A	0.9920635	KDM5C	Nonsyn	R943*
HN137-Met-cell-line	chr3	138461535	T	W	0.4515245	PIK3CB	Syn_or_UTR	-
HN137-Met-cell-line	chr4	66242766	G	K	0.572139	EPHA5	Syn_or_UTR	-
HN137-Met-cell-line	chr8	57079885	C	Y	0.1917125	PLAG1	Syn_or_UTR	-
HN148-Met-Hu	chr17	7578266	T	W	0.2674135	TP53	Nonsyn	I195F
HN148-Met-Hu	chr8	93003941	G	R	0.2403705	RUNX1T1	Nonsyn	A317V

HN148-Met-Hu	chr3	186502455	C	S	0.129118	EIF4A2	Nonsyn	Q60E
HN148-Met-Hu	chr19	11105568	G	R	0.1200195	SMARCA4	Nonsyn	G495D
HN148-Met-Hu	chr3	38595822	G	R	0.1194845	SCN5A	Syn_or_UTR	-
HN148-Met-Hu	chr6	134495172	C	S	0.108711	SGK1	Nonsyn	E162Q
HN120-Met-Hu	chr17	7577142	C	Y	0.2493615	TP53	Nonsyn	G266R
HN120-Met-Hu	chr17	7577538	C	Y	0.2263315	TP53	Nonsyn	R248Q
HN120-Met-Hu	chr10	89692905	G	R	0.224497	PTEN	Nonsyn	R130Q
HN120-Met-Hu	chr9	21970971	G	S	0.6300655	CDKN2A	Nonsyn	Y129*
HN120-Met-Hu	chr19	40901505	C	Y	0.4059085	PRX	Syn_or_UTR	-
HN120-Met-Hu	chr14	105240204	G	S	0.2724865	AKT1	Syn_or_UTR	-
HN120-Met-Hu	chr10	76748854	G	R	0.2826885	KAT6B	Syn_or_UTR	-
HN120-Met-Hu	chr16	9858531	C	Y	0.25883	GRIN2A	Nonsyn	G957E
HN120-Met-Hu	chr22	23540444	G	R	0.237274	BCR	Syn_or_UTR	-
HN120-Met-Hu	chr16	9857975	G	K	0.2099475	GRIN2A	Syn_or_UTR	-
HN120-Met-Hu	chr9	80646189	C	Y	0.167419	GNAQ	Syn_or_UTR	-
HN159-Met-Hu	chr17	7577124	C	M	0.180627	TP53	Nonsyn	V272L
HN159-Met-Hu	chr17	76993389	G	R	0.16831	CANT1	Nonsyn	R106*
HN159-Met-Hu	chr2	85419169	G	R	0.1638685	TCF7L1	Intron_or_UTR	-
HN159-Met-Hu	chr3	121206503	G	K	0.1413275	POLQ	Nonsyn	P1759T
HN159-Met-Hu	chr14	75022279	G	R	0.141894	LTBP2	Syn_or_UTR	-
HN159-Met-Hu	chrX	102754975	C	Y	0.13113	RAB40A	Nonsyn	R237Q
HN159-Met-Hu	chr2	80846308	T	Y	0.125	CTNNA2	Syn_or_UTR	-
HN159-Met-Hu	chr11	118347634	C	Y	0.109712	MLL	Nonsyn	P1091S
HN159-Met-Hu	chr15	66777347	C	S	0.102239	MAP2K1	Nonsyn	T238S
HN148-Pri-Hu	chr17	7578266	T	W	0.1891275	TP53	Nonsyn	I195F
HN148-Pri-Hu	chr8	93003941	G	R	0.1438055	RUNX1T1	Nonsyn	A317V
HN148-Pri-Hu	chr19	11105568	G	R	0.1176625	SMARCA4	Nonsyn	G495D
HN120-Pri-Hu	chr17	7577538	C	Y	0.289425	TP53	Nonsyn	R248Q
HN120-Pri-Hu	chr10	89692905	G	R	0.2940995	PTEN	Nonsyn	R130Q
HN120-Pri-Hu	chr17	7577142	C	Y	0.2627925	TP53	Nonsyn	G266R
HN120-Pri-Hu	chr9	21970971	G	S	0.676909	CDKN2A	Nonsyn	Y129*

HN120-Pri-Hu	chr17	63530200	G	R	0.282877	AXIN2	possible_3_prime_splice	-
HN120-Pri-Hu	chr2	202136259	C	Y	0.2775625	CASP8	Nonsyn	S168L
HN120-Pri-Hu	chr22	36708219	G	R	0.266273	MYH9	Nonsyn	P535S
HN120-Pri-Hu	chr10	76748854	G	R	0.256585	KAT6B	Syn_or_UTR	-
HN120-Pri-Hu	chr14	105240204	G	S	0.2497215	AKT1	Syn_or_UTR	-
HN120-Pri-Hu	chr16	9857975	G	K	0.250373	GRIN2A	Syn_or_UTR	-
HN120-Pri-Hu	chr6	134492137	G	R	0.238627	SGK1	Syn_or_UTR	-
HN120-Pri-Hu	chr9	80646189	C	Y	0.206215	GNAQ	Syn_or_UTR	-
HN137-Pri-PDX	chr1	65332664	A	W	0.437714	JAK1	Nonsyn	L292Q
HN137-Pri-PDX	chr10	70332852	C	Y	0.4391475	TET1	Nonsyn	P253S
HN137-Pri-PDX	chr17	7578176	C	T	1	TP53	Nonsyn	V186I
HN137-Pri-PDX	chr21	42869119	G	K	0.10070815	TMPRSS2	Intron_or_UTR	-
HN137-Pri-PDX	chrX	53226022	G	A	1	KDM5C	Nonsyn	R943*
HN137-Pri-PDX	chr2	190717464	C	Y	0.236588	PMS1	Syn_or_UTR	-
HN137-Pri-PDX	chr3	138461535	T	W	0.523995	PIK3CB	Syn_or_UTR	-
HN137-Pri-PDX	chr4	66242766	G	K	0.482069	EPHA5	Syn_or_UTR	-
HN137-Pri-Hu	chr1	65332664	A	W	0.2592495	JAK1	Nonsyn	L292Q
HN137-Pri-Hu	chr10	70332852	C	Y	0.2526785	TET1	Nonsyn	P253S
HN137-Pri-Hu	chr17	7578176	C	T	0.4243815	TP53	Nonsyn	V186I
HN137-Pri-Hu	chrX	53226022	G	A	0.48258	KDM5C	Nonsyn	R943*
HN137-Pri-Hu	chr2	190717464	C	Y	0.113784	PMS1	Syn_or_UTR	-
HN137-Pri-Hu	chr3	138461535	T	W	0.2318805	PIK3CB	Syn_or_UTR	-
HN137-Pri-Hu	chr4	66242766	G	K	0.26447	EPHA5	Syn_or_UTR	-
HN137-Met-PDX	chr1	65332664	A	W	0.4470295	JAK1	Nonsyn	L292Q
HN137-Met-PDX	chr10	70332852	C	Y	0.5140055	TET1	Nonsyn	P253S
HN137-Met-PDX	chr17	7578176	C	T	0.998457	TP53	Nonsyn	V186I
HN137-Met-PDX	chrX	53226022	G	A	0.985513	KDM5C	Nonsyn	R943*
HN137-Met-PDX	chr3	138461535	T	W	0.4643805	PIK3CB	Syn_or_UTR	-
HN137-Met-PDX	chr4	66242766	G	K	0.271085	EPHA5	Syn_or_UTR	-
HN137-Pri-cell-line	chr1	65332664	A	W	0.452333	JAK1	Nonsyn	L292Q
HN137-Pri-cell-line	chr10	70332852	C	Y	0.575653	TET1	Nonsyn	P253S

HN137-Pri-cell-line	chr17	7578176	C	T	1	TP53	Nonsyn	V186I
HN137-Pri-cell-line	chrX	53226022	G	A	0.9529145	KDM5C	Nonsyn	R943*
HN137-Pri-cell-line	chr2	190717464	C	Y	0.3674325	PMS1	Syn_or_UTR	-
HN137-Pri-cell-line	chr3	138461535	T	W	0.3645965	PIK3CB	Syn_or_UTR	-
HN137-Pri-cell-line	chr4	66242766	G	K	0.609796	EPHA5	Syn_or_UTR	-
HN137-Met-Hu	chr1	65332664	A	W	0.240304	JAK1	Nonsyn	L292Q
HN137-Met-Hu	chr10	70332852	C	Y	0.1867025	TET1	Nonsyn	P253S
HN137-Met-Hu	chr14	102549685	T	Y	0.10541735	HSP90AA1	Intron_or_UTR	-
HN137-Met-Hu	chr17	7578176	C	T	0.237135	TP53	Nonsyn	V186I
HN137-Met-Hu	chrX	53226022	G	A	0.3889705	KDM5C	Nonsyn	R943*
HN137-Met-Hu	chr3	138461535	T	W	0.183483	PIK3CB	Syn_or_UTR	-
HN137-Met-Hu	chr4	66242766	G	K	0.2269005	EPHA5	Syn_or_UTR	-

Supplementary Table 2

POLARIS Xplora panel

ABCA1	CDH1	EXT2	IRS2	NBN	RAD51	STAT4
ABI1	CDH10	EZH1	ITGA10	NBPF1	RAD51B	STIL
ABL1	CDH11	EZH2	ITGA9	NCOA1	RADIL	STK11
ABL2	CDH2	FAM135B	ITGB3	NCOA2	RAF1	STK36
ACKR3	CDH20	FAM46C	ITK	NCOA4	RALGDS	SUFU
ACSL6	CDH5	FAM58A	JAK1	NCOR1	RANBP17	SUZ12
ACTB	CDH6	FANCA	JAK2	NCOR2	RAP1GDS1	SYK
ACVR2A	CDK12	FANCC	JAK3	NDC80	RARA	SYNE1
ADAM29	CDK4	FANCD2	JARID2	NDRG1	RASA1	TACC3
ADAMTS20	CDK6	FANCE	JUN	NF1	RB1	TAF1
ADGRA2	CDK8	FANCF	KAT6A	NF2	RBM10	TAF1L
ADGRB3	CDKN1A	FANCG	KAT6B	NFE2L2	RBM15	TAL1
ADGRL3	CDKN1B	FANCL	KDM2B	NFKB1	RECQL4	TBL1XR1
AFF1	CDKN2A	FAS	KDM5A	NFKB2	REL	TBX22
AFF3	CDKN2B	FAT1	KDM5B	NFKBIA	RELN	TBX3
AKAP9	CDKN2C	FAT4	KDM5C	NIN	RET	TBX5
AKT1	CDX2	FBXO11	KDM6A	NKX2-1	RHOA	TCF12
AKT2	CEBPA	FBXW7	KDR	NLRP1	RHOH	TCF3
AKT3	CHD2	FCRL4	KEAP1	NONO	RICTOR	TCF7L1
ALDH2	CHD4	FGF10	KEL	NOTCH1	RIT1	TCF7L2
ALK	CHD5	FGF14	KIAA1549	NOTCH2	RMI2	TCL1A
ALPK2	CHD6	FGF19	KIF1B	NOTCH3	RNASEL	TERT
AMER1	CHD8	FGF23	KIT	NOTCH4	RNF2	TET1
ANK3	CHEK1	FGF6	KL	NPM1	RNF213	TET2
APC	CHEK2	FGFR1	KLF5	NR4A3	RNF43	TFE3
AR	CHIC2	FGFR2	KLF6	NRAS	ROBO2	TFPT
ARAF	CIC	FGFR3	KLHL6	NSD1	ROS1	TGFBR2
ARHGAP26	CIITA	FGFR4	KMT2A	NT5C2	RPL22	TGM7
ARHGAP35	CKS1B	FH	KMT2B	NTRK1	RPS6KA2	THBS1
ARHGEF12	CLTC	FHIT	KMT2C	NTRK2	RPTOR	TIAM1
ARID1A	CLTCL1	FIP1L1	KMT2D	NTRK3	RRM1	TIMP3
ARID1B	CMPK1	FLCN	KRAS	NUMA1	rs10411210	TLR4
ARID2	CNTRL	FLI1	KTN1	NUP210L	rs10929302	TLX1
ARID4A	COL1A1	FLT1	LAMA2	NUP214	rs4124874	TMPRSS2
ARID4B	COL5A1	FLT3	LAMP1	NUP93	rs10936599	TNFAIP3
ARID5A	CPS1	FLT4	LASP1	NUP98	rs11045585	TNFRSF14
ARID5B	CRBN	FLYWCH1	LCK	NUTM1	RS11169552	TNFRSF17
ARNT	CREB1	FN1	LIFR	OR4A16	rs1321311	TNK2
ASH1L	CREB3L1	FOXL2	LMO1	OTUD7A	rs1665650	TNKS2
ASPSCR1	CREB3L2	FOXO1	LMO2	P2RY8	rs1728785	TOP1
ASXL1	CREBBP	FOXO3	LPP	PAIP1	rs17868323	TOP2A

ATF1	CRKL	FOXO4	LRP1B	PAK3	rs2423279	TOP2B
ATIC	CRLF2	FOXP1	LRP5	PAK7	rs3824999	TP53
ATM	CRTC1	FOXP4	LRP6	PALB2	rs4444235	TP63
ATR	CSF1R	FRS2	LRRK2	PALLD	rs4925386	TPM3
ATRX	CSF3R	FUBP1	LTBP2	PARP1	rs5934683	TPM4
AURKA	CSMD3	FUS	LTF	PATZ1	rs6017342	TPR
AURKB	CTCF	FZD10	LTK	PAX3	rs647161	TPX2
AURKC	CTNNA1	FZR1	LYL1	PAX5	rs6687758	TRAF3
AXIN1	CTNNA2	G6PD	MAF	PAX7	rs6691170	TRIM24
AXIN2	CTNNB1	GAS7	MAFB	PAX8	rs6983267	TRIM27
AXL	CTNND2	GATA1	MAGEA1	PBRM1	rs7586110	TRIM33
B2M	CUX1	GATA2	MAGI1	PBX1	rs886774	TRIP11
BAP1	CYLD	GATA3	MALAT1	PCBP1	rs961253	TRRAP
BARD1	CYP2C19	GDNF	MALT1	PCDHA13	rs9929218	TSC1
BAZ2B	CYP2D6	GID4	MAML2	PCM1	RSPO2	TSC2
BCL10	DAAM2	GLI3	MAP2K1	PDCD11	RSPO3	TSHR
BCL11A	DAXX	GNA11	MAP2K2	PDCD1LG2	RUNX1	TSHZ2
BCL11B	DCC	GNA13	MAP2K4	PDE4DIP	RUNX1T1	TSHZ3
BCL2	DCLK1	GNAQ	MAP3K1	PDGFB	RXRA	TTLL9
BCL2L1	DDB2	GNAS	MAP3K14	PDGFRA	SAMD9	TXNIP
BCL2L2	DDIT3	GNPTAB	MAP3K6	PDGFRB	SARDH	TYK2
BCL3	DDR2	GOPC	MAP3K7	PDK1	SBDS	U2AF1
BCL6	DDX10	GPC3	MAP3K8	PEG3	SCN5A	U2AF2
BCL7A	DDX3X	GPHN	MAPK1	PER1	SDHA	UBR5
BCL9	DDX5	GRIN2A	MAPK8	PGAP3	SDHAF2	UGT1A1
BCLAF1	DEK	GRM8	MARK1	PHF6	SDHB	USP6
BCOR	DICER1	GSK3B	MARK4	PHOX2B	SDHC	USP9X
BCORL1	DLC1	GTSE1	MAX	PIK3C2B	SDHD	UTY
BCR	DNER	GUCY1A2	MBD1	PIK3CA	40057	VHL
BIRC2	DNMT3A	HCAR1	MCL1	PIK3CB	SETBP1	VTI1A
BIRC3	DOCK2	HDAC4	MDM2	PIK3CD	SETD1B	WAS
BIRC5	DOT1L	HECW1	MDM4	PIK3CG	SETD2	WASF3
BLM	DPYD	HEY1	MECOM	PIK3R1	SETDB1	WDR90
BLNK	DST	HGF	MED12	PIK3R2	SF3A1	WHSC1
BMPR1A	E2F1	HIF1A	MEF2B	PIM1	SF3B1	WHSC1L1
BRAF	EBF1	HIP1	MEN1	PKHD1	SF3B2	WIF1
BRCA1	EGFR	HIST1H1C	MET	PLAG1	SGK1	WISP3
BRCA2	EIF4A2	HIST1H1E	MITF	PLCG1	SH2D1A	WRN
BRD3	ELF3	HIST1H2AM	MKL1	PLCG2	SIN3A	WT1
BRD4	EML4	HIST1H3B	MLH1	PLEKHG5	SLC4A5	XIRP2
BRD8	EP300	HLA-A	MLLT10	PLK1	SLIT2	XPA
BRIP1	EP400	HLF	MLLT4	PML	SMAD2	XPC
BTG1	EPCAM	HMGA1	MMP2	PMS1	SMAD3	XPO1

BTG2	EPHA3	HMGA2	MN1	PMS2	SMAD4	XRCC2
BTK	EPHA5	HNF1A	MORC4	POLD1	SMARCA1	YAP1
BUB1B	EPHA6	HNRNPA1	MPL	POLE	SMARCA2	ZFHX3
C11orf30	EPHA7	HOOK3	MRE11A	POLQ	SMARCA4	ZFP36L1
CANT1	EPHB1	HOXA11	MSH2	POT1	SMARCB1	ZIC4
CAP2	EPHB4	HOXC13	MSH3	POU2AF1	SMARCC1	ZMYM2
CARD11	EPHB6	HOXD13	MSH6	POU5F1	SMC1A	ZMYM3
CARS	EPPK1	HRAS	MTCP1	PPARG	SMC3	ZNF217
CASC5	ERBB2	HSP90AA1	MTOR	PPP2R1A	SMG1	ZNF331
CASP8	ERBB3	HSP90AB1	MTR	PRCC	SMO	ZNF384
CBFA2T3	ERBB4	ICK	MTRR	PRDM1	SMUG1	ZNF521
CBFB	ERC1	IDH1	MTUS2	PRDM16	SNX25	ZNF703
CBL	ERCC1	IDH2	MUC1	PRF1	SOCS1	ZNRF3
CBLB	ERCC2	IGF1R	MUC17	PRKAR1A	SOS1	ZRSR2
CCDC6	ERCC3	IGF2	MUC6	PRKDC	SOX10	
CCND1	ERCC4	IGF2R	MUTYH	PRRX1	SOX11	
CCND2	ERCC5	IKBKB	MYB	PRX	SOX2	
CCND3	ERCC6	IKBKE	MYC	PSIP1	SPEN	
CCNE1	ERG	IKZF1	MYCL	PTCH1	SPOP	
CD1D	ESR1	IL2	MYCN	PTEN	SRC	
CD274	ETS1	IL21R	MYD88	PTGS2	SRGAP3	
CD70	ETV1	IL6ST	MYH11	PTPN11	SRSF1	
CD74	ETV4	IL7R	MYH9	PTPRD	SRSF2	
CD79A	ETV5	ING4	MYOCD	PTPRT	SRSF3	
CD79B	ETV6	INHBA	NALCN	RAB40A	SSX1	
CDC27	EWSR1	INPPL1	NAV2	RAD21	STAG2	
CDC73	EXT1	IRF4	NAV3	RAD50	STAT3	

Supplementary Table 3

MSIG analysis of genes upregulated in HN137-Met versus HN137-Pri

Pathway	P-value	FDR-correct P-value
HALLMARK_INTERFERON_GAMMA_RESPONSE	0.00	0.00
HALLMARK_EPITHELIAL_MESENCHYMAL_TRANSITION	0.00	0.00
HALLMARK_INTERFERON_ALPHA_RESPONSE	0.00	0.00
HALLMARK_TNFA_SIGNALING_VIA_NFKB	0.00	0.00
HALLMARK_INFLAMMATORY_RESPONSE	0.00	0.00
NABA_MATRISOME	0.00	0.00
REACTOME_INTERFERON_ALPHA_BETA_SIGNALING	0.00	0.00
Genes_Up-Regulated_During_EMT	0.00	0.00
HALLMARK_UV_RESPONSE_DN	0.00	0.00
NABA_CORE_MATRISOME	0.00	0.00
HALLMARK_KRAS_SIGNALING_UP	0.00	0.00
REACTOME_INTERFERON_SIGNALING	0.00	0.00
HALLMARK_ALLOGRAFT_REJECTION	0.00	0.00
EMT	0.00	0.00
HALLMARK_COMPLEMENT	0.00	0.00
NABA_ECM_GLYCOPROTEINS	0.00	0.00
REACTOME_INTERFERON_GAMMA_SIGNALING	0.00	0.00
KEGG_CYTOKINE_CYTOKINE_RECEPTOR_INTERACTION	0.00	0.00
HALLMARK_IL6_JAK_STAT3_SIGNALING	0.00	0.00
NABA_MATRISOME_ASSOCIATED	0.00	0.00
HALLMARK_COAGULATION	0.00	0.00
REACTOME_CYTOKINE_SIGNALING_IN_IMMUNE_SYSTEM	0.00	0.00
PID_INTEGRIN1_PATHWAY	0.00	0.00
HALLMARK_IL2_STAT5_SIGNALING	0.00	0.00
KEGG_ECM_RECEPTOR_INTERACTION	0.00	0.00
REACTOME_RESPONSE_TO_ELEVATED_PLATELET_CYTOSOLIC_CA2_	0.00	0.00
HALLMARK_APOPTOSIS	0.00	0.00
HALLMARK_HYPOXIA	0.00	0.00
KEGG_NOD_LIKE_RECEPTOR_SIGNALING_PATHWAY	0.00	0.00
REACTOME_EXTRACELLULAR_MATRIX_ORGANIZATION	0.00	0.00
HALLMARK_ANGIOGENESIS	0.00	0.00
REACTOME_ANTIGEN_PRESENTATION_FOLDING_ASSEMBLY_AND_PEPTIDE_LOADING_OF_CLASS_I_MHC	0.00	0.00
PID_AMB2_NEUTROPHILS_PATHWAY	0.00	0.00
NABA_ECM_REGULATORS	0.00	0.00
PID_SYNDECAN_1_PATHWAY	0.00	0.00
PID_AVB3_INTEGRIN_PATHWAY	0.00	0.01
BIOCARTA_INFLAM_PATHWAY	0.00	0.01
PID_INTEGRIN3_PATHWAY	0.00	0.01

PID_SYNDECAN_4_PATHWAY	0.00	0.01
REACTOME_ENDOSOMAL_VACUOLAR_PATHWAY	0.00	0.01
KEGG_TOLL_LIKE_RECEPTOR_SIGNALING_PATHWAY	0.00	0.01
REACTOME_IMMUNOREGULATORY_INTERACTIONS_BETWEEN_A_LYMPHOID_AND_A_NON_LYMPHOID_CELL	0.00	0.01
NABA_SECRETED_FACTORS	0.00	0.01
BIOCARTA_LAIR_PATHWAY	0.00	0.01
REACTOME_AMYLOIDS	0.00	0.01
BIOCARTA_GRANULOCYTES_PATHWAY	0.00	0.01
KEGG_TYPE_I_DIABETES_MELLITUS	0.00	0.02
KEGG_CELL_ADHESION_MOLECULES_CAMS	0.00	0.02
PID_IL23_PATHWAY	0.00	0.03
REACTOME_IMMUNE_SYSTEM	0.00	0.03
REACTOME_NEGATIVE_REGULATORS_OF_RIG_I_MDA5_SIGNALING	0.00	0.03
KEGG_NATURAL_KILLER_CELL_MEDIATED_CYTOTOXICITY	0.00	0.04
REACTOME_PACKAGING_OF_TELOMERE_ENDS	0.00	0.04
KEGG_COMPLEMENT_AND_COAGULATION_CASCADES	0.00	0.04
REACTOME_RIG_I_MDA5_MEDIATED_INDUCTION_OF_IFN_ALPHA_BETA_PATHWAYS	0.00	0.04
KEGG_GRAFT_VERSUS_HOST_DISEASE	0.00	0.04

Supplementary Table 4

MSIG analysis of genes downregulated in HN137-Met versus HN137-Pri

Pathway	P-value	FDR-correct P-value
AIGNER_ZEB1_TARGETS	0.00	0.00
HALLMARK_ESTROGEN_RESPONSE_LATE	0.00	0.00
NABA_MATRISOME	0.00	0.00
NABA_MATRISOME_ASSOCIATED	0.00	0.00
HALLMARK_ESTROGEN_RESPONSE_EARLY	0.00	0.00
HALLMARK_KRAS_SIGNALING_DN	0.00	0.00
REACTOME_BIOLOGICAL_OXIDATIONS	0.00	0.00
NABA_SECRETED_FACTORS	0.00	0.00
KEGG_STEROID_HORMONE_BIOSYNTHESIS	0.00	0.01
REACTOME_PHASE1_FUNCTIONALIZATION_OF_COMPOUNDS	0.00	0.01
HALLMARK_APICAL_JUNCTION	0.00	0.01
HALLMARK_P53_PATHWAY	0.00	0.01
KEGG_METABOLISM_OF_XENOBIOTICS_BY_CYTOCHROME_P450	0.00	0.01
NABA_ECM_REGULATORS	0.00	0.01
KEGG_RETINOL_METABOLISM	0.00	0.04
REACTOME_BMAL1_CLOCK_NPAS2_ACTIVATES_CIRCADIAN_EXPRESSION	0.00	0.04
NABA_ECM_AFFILIATED	0.00	0.04

Supplementary Table 5

List of qPCR primer sequences

Gene	Sequence of primer pair
CDH1	5' CGAGAGCTACACGTTCACGG 3'
	5' GGGTGTCGAGGGAAAAATAGG 3'
CLDN4	5' TGGGGCTACAGGTAATGGG 3'
	5' GGTCTGCGAGGTGACAATGTT 3'
CLDN17	5' CTTGGCATGGTGGGGACTC 3'
	5' CTGGCTTGTCGGATGCAATTC 3'
ZEB1	5' TTACACCTTTGCATACAGAACCC 3'
	5' TTTACGATTACACCCAGACTGC 3'
ZEB2	5' CAAGAGGCGCAAACAAGCC 3'
	5' GGTTGGCAATACCGTCATCC 3'
VIM	5' GACGCCATCAACACCGAGTT
	5' CTTTGTCGTTGGTTAGCTGGT 3'
SNAI1	5' ACTGCAACAAGGAATACCTCAG 3'
	5' GCACTGGTACTTCTTGACATCTG 3
FN1	5' CGGTGGCTGTCAGTCAAAG
	5' AAACCTCGGCTTCCTCCATAA 3'
SERPINE1	5' ACCGCAACGTGGTTTTCTCA 3'
	5' TTGAATCCCATAGCTGCTTGAAT 3'
AMOTL2	5' TGCGAGAGAAGGAGGAGCAGA 3'
	5' GGGCATGGAGCACCTTTAACC 3'
CTGF	5' CACCCGGGTTACCAATGACAA 3'
	5' GGTGCAGCCAGAAAGCTCAAA 3'
CYR61	5' AGGGCAAGAAATGCAGCAAGA 3'
	5' CGGAACCGCATCTTCACAGTC 3'
YAP1	5' TGAACGTCACAGCCCCCACC 3'
	5' ACGAGGGTCAAGCCTTGGGTC 3'

Supplementary Table 6

List of compounds in SelleckChem anti-cancer library

ABT-263 (Navitoclax)	BMS-599626 (AC480)	XL147
ABT-737	Obatoclax mesylate (GX15-070)	Everolimus (RAD001)
Linifanib (ABT-869)	Olaparib (AZD2281)	TW-37
ABT-888 (Veliparib)	Nutlin-3	Mocetinostat (MGCD0103)
Axitinib	Masitinib (AB1010)	Abiraterone (CB-7598)
Saracatinib (AZD0530)	GDC-0941	SRT1720
AZD6244 (Selumetinib)	SB 431542	YM155
BEZ235 (NVP-BEZ235)	Crizotinib (PF-02341066)	MLN8237 (Alisertib)
BIBF1120 (Vargatef)	AUY922 (NVP-AUY922)	AT9283
Afatinib (BIBW2992)	PHA-665752	Pemetrexed (Alimta)
Bortezomib (Velcade)	ZSTK474	Andarine (GTX-007)
Bosutinib (SKI-606)	SB 216763	17-AAG (Tanespimycin)
Cediranib (AZD2171)	SB 203580	17-DMAG HCl (Alvespimycin)
Dovitinib (TKI-258)	MK-2206 2HCl	SNS-032 (BMS-387032)
CI-1040 (PD184352)	PD153035 HCl	Cyclopamine
Dasatinib (BMS-354825)	SU11274	Barasertib (AZD1152-HQPA)
Deforolimus (Ridaforolimus)	Vismodegib (GDC-0449)	Docetaxel (Taxotere)
Erlotinib HCl	Brivanib (BMS-540215)	Gemcitabine HCl (Gemzar)
Gefitinib (Iressa)	Belinostat (PXD101)	Paclitaxel (Taxol)
Imatinib Mesylate	Iniparib (BSI-201)	Roscovitine (Seliciclib, CYC202)
Lapatinib Ditosylate (Tykerb)	PCI-24781	SNS-314 Mesylate
Lenalidomide (Revlimid)	Linsitinib (OSI-906)	Capecitabine (Xeloda)
Motesanib Diphosphate	KU-55933	Ganetespib (STA-9090)
Nilotinib (AMN-107)	GSK1904529A	E7080 (Lenvatinib)
PD0325901	PF-04217903	ABT-751
PI-103	JNJ-26481585	Cisplatin
Rapamycin (Sirolimus)	BTZ043 racemate	Valproic acid sodium salt
Sorafenib (Nexavar)	Rucaparib	CYC116
STF-62247	Vatalanib 2HCl (PTK787)	JNJ 26854165 (Serdemetan)
Sunitinib Malate (Sutent)	GDC-0879	WZ4002
Tandutinib (MLN518)	LY294002	Ostarine (MK-2866)
Temsirolimus (Torisel)	Danusertib (PHA-739358)	BIIB021
Trichostatin A (TSA)	TAE684 (NVP-TAE684)	Regorafenib (BAY 73-4506)
Vandetanib (Zactima)	BI 2536	XAV-939
Vorinostat (SAHA)	SGX-523	ENMD-2076
VX-680 (MK-0457, Tozasertib)	GSK690693	BIBR 1532
Y-27632 2HCl	JNJ-38877605	PIK-90
Elesclomol	PD 0332991 (Palbociclib) HCl	Anastrozole
Entinostat	Triciribine (Triciribine phosphate)	Aprepitant (MK-0869)
Enzastaurin (LY317615)	Fingolimod (FTY720)	Bicalutamide (Casodex)
Fulvestrant (Faslodex)	MDV3100 (Enzalutamide)	AT7519
Raltitrexed (Tomudex)	Celecoxib	MK-1775
Thalidomide	PD173074	Quizartinib (AC220)
CUDC-101	WYE-354	AZD7762
Exemestane	Vemurafenib (PLX4032)	R406 (free base)
Irinotecan	IC-87114	DMXAA (ASA404)
Cladribine	BX-795	EX 527
Decitabine	Altretamine (Hexalen)	Febuxostat (Uloric)

Dimesna	Carmofur	Dapagliflozin
PIK-75	Epothilone A	AZD8055
Tivozanib (AV-951)	Floxuridine (Fludara)	BMS 777607
Doxorubicin (Adriamycin)	Ftorafur	Pomalidomide
Adrucil (Fluorouracil)	Ifosfamide	KU-60019
Abitrexate (Methotrexate)	Megestrol Acetate	BIRB 796 (Doramapimod)
Imiquimod	Mercaptopurine	Tie2 kinase inhibitor
Bendamustine HCL	Pamidronate Disodium	Ubenimex (Bestatin)
Nelarabine (Arranon)	Streptozotocin (Zanosar)	Prednisone (Adasone)
Bleomycin sulfate	Dexamethasone	Triamcinolone Acetonide
Carboplatin	Rigosertib (ON-01910)	Cytarabine
Clafen (Cyclophosphamide)	Epothilone B (EPO906)	Tretinoin (Aberela)
Clofarabine	Dorzolamide HCl	Ezetimibe (Zetia)
YM201636	Ruxolitinib (INCB018424)	Estrone
OSI-930	Isotretinoin	Aminoglutethimide (Cytadren)
Dacarbazine (DTIC-Dome)	Pelitinib (EKB-569)	Disulfiram (Antabuse)
Epirubicin HCl	Zileuton	Betapar (Meprednisone)
Oxaliplatin (Eloxatin)	Ispinesib (SB-715992)	Busulfan (Myleran, Busulfex)
Etoposide (VP-16)	Tipifarnib (Zarnestra)	Hydrocortisone (Cortisol)
Ku-0063794	Zibotentan (ZD4054)	Estradiol
Evista (Raloxifene HCl)	Doxercalciferol (Hectorol)	Gemcitabine (Gemzar)
Idarubicin HCl	SB 525334	Azathioprine (Azasan, Imuran)
Fludarabine Phosphate	AEE788 (NVP-AEE788)	Mesna (Uromitexan, Mesnex)
Topotecan HCl	PHA-793887	Toremifene Citrate
2-Methoxyestradiol	PIK-93	Azacitidine (Vidaza)
Letrozole	Ponatinib (AP24534)	Teniposide (Vumon)
Leucovorin Calcium	Fludarabine (Fludara)	Simvastatin (Zocor)
Temozolomide	LY2228820	Ranolazine (Ranexa)
Vincristine	Mycophenolate mofetil	Lomustine (CeeNU)
Amuvatinib (MP-470)	SB939 (Pracinostat)	D-glutamine
Vinblastine	Tosedostat (CHR2797)	Hydroxyurea (Cytodrox)
JNJ-7706621	XL765 (SAR245409)	Flutamide (Eulexin)
Fluvastatin sodium (Lescol)	Phloretin (Dihydronaringenin)	TG101348 (SAR302503)
Tamoxifen Citrate (Nolvadex)	Salinomycin (Procoxacin)	PAC-1
Procarbazine HCl (Matulane)	Quercetin (Sophoretin)	AZ628
Sodium butyrate	Coenzyme Q10 (CoQ10)	AT-406
Maraviroc	Chrysophanic acid	Canagliflozin
PF 573228	Imatinib (Gleevec)	3-Methyladenine
Cyclophosphamide monohydrate	Itraconazole (Sporanox)	Dalcetrapib (JTT-705)
Bexarotene	Mitoxantrone HCl	Nocodazole
Vinpocetine (Cavinton)	Mycophenolic (Mycophenolate)	GW4064
Lapatinib	Rosiglitazone (Avandia)	Tofacitinib
Neratinib (HKI-272)	Medroxyprogesterone acetate	Sotrastaurin (AEB071)
LDE225	Pioglitazone (Actos)	APO866 (FK866)
AG14361	Mifepristone (Mifeprex)	Sirtinol
MLN2238	Lonidamine	CEP33779
MLN9708	TAK-733	INK 128 (MLN0128)
SB 743921	LDN193189	Torin 2
GSK461364	LY2603618 (IC-83)	RG108
SGI-1776 free base	GW3965 HCl	TPCA-1

BMS 794833	DCC-2036 (Rebastinib)	Desmethyl Erlotinib (CP-473420)
OSI-420	NU7441 (KU-57788)	Torin 1
R935788	GSK2126458	PF-562271
Formestane	MK-0752	S-Ruxolitinib
DAPT (GSI-IX)	PF-3845	BAY 11-7082 (BAY 11-7821)
Irinotecan HCl Trihydrate	GSK1120212 (Trametinib)	CHIR-99021 (CT99021) HCl
Cyt387	Flavopiridol (Alvocidib) HCl	Pazopanib
SB590885	PCI-32765 (Ibrutinib)	Daunorubicin HCl
TAME	NVP-BSK805 2HCl	Dexamethasone acetate
CAL-101 (GS-1101)	WAY-362450	Anagrelide HCl
LY2157299	A-769662	Triptolide
Telatinib (BAY 57-9352)	CH5132799	AZ 3146
BI6727 (Volasertib)	KX2-391	Gossypol
Palomid 529	LY2109761	Crenolanib (CP-868596)
WP1130	YO-01027	Cyclosporin A (Cyclosporine A)
AR-42 (HDAC-42)	Geldanamycin	Dacomitinib
CP-466722	AMG 900	(-)-Epigallocatechin gallate
BKM120 (NVP-BKM120)	PF-03814735	
CX-4945 (Silmitasertib)	PH-797804	

Supplementary Table 7

List of compounds in SelleckChem kinase inhibitor library

Linifanib (ABT-869)	Tivozanib (AV-951)	TAK-733
Axitinib	YM201636	LDN193189
Saracatinib (AZD0530)	OSI-930	AZD5438
AZD6244 (Selumetinib)	Ku-0063794	PP-121
BEZ235 (NVP-BEZ235)	AG-1024	OSI-027
BIBF1120 (Vargatef)	Amuvatinib (MP-470)	R788 (Fostamatinib)
Afatinib (BIBW2992)	JNJ-7706621	LY2603618 (IC-83)
Bosutinib (SKI-606)	PD173074	PF-05212384 (PKI-587)
Cediranib (AZD2171)	WYE-354	DCC-2036 (Rebastinib)
Dovitinib (TKI-258)	Vemurafenib (PLX4032)	CCT128930
CI-1033 (Canertinib)	IC-87114	A66
CI-1040 (PD184352)	BX-795	NU7441(KU-57788)
Dasatinib (BMS-354825)	BX-912	GSK2126458
Deforolimus (Ridaforolimus)	AMG-208	WYE-125132
Erlotinib HCl	TG100-115	WYE-687
Gefitinib (Iressa)	GSK1059615	A-674563
Imatinib Mesylate	MGCD-265	AS-252424
Lapatinib Ditosylate (Tykerb)	ON-01910	PF-00562271
Motesanib Diphosphate	Ki8751	GSK1120212 (Trametinib)
Nilotinib (AMN-107)	Ruxolitinib (INCB018424)	Flavopiridol hydrochloride
Pazopanib HCl	Pelitinib (EKB-569)	PCI-32765 (Ibrutinib)
PD0325901	AS-605240	AS-604850
PI-103	Staurosporine	CAY10505
Rapamycin (Sirolimus)	Aurora A Inhibitor I	CHIR-124
Sorafenib (Nexavar)	PHA-680632	NVP-BSK805
Sunitinib Malate (Sutent)	Thiazovivin	WAY-600
Tandutinib (MLN518)	SP600125	TG101209
Temsirolimus (Torisel)	TSU-68	GDC-0980 (RG7422)
Vandetanib (Zactima)	AS703026	A-769662
VX-680 (MK-0457, Tozasertib)	SB 525334	KX2-391
Y-27632 2HCl	HMN-214	GSK1838705A
Enzastaurin (LY317615)	AEE788 (NVP-AEE788)	TAK-901
BMS-599626 (AC480)	PHA-793887	AMG 900
Masitinib (AB1010)	PIK-93	ZM 336372
GDC-0941	Ponatinib (AP24534)	PF-03814735
SB 431542	LY2228820	PH-797804
Crizotinib (PF-02341066)	CCT129202	Dacomitinib
PHA-665752	XL765	AG-1478 (Tyrphostin AG-1478)
ZSTK474	AT7519	SB 415286
SB 216763	Quizartinib (AC220)	Crenolanib (CP-868596)
SB 203580	Hesperadin	TG101348 (SAR302503)
SB 202190	BIX 02188	PKI-402
MK-2206 dihydrochloride	BIX 02189	GSK1070916
PD153035 HCl	AZD7762	PHA-767491
SU11274	R406(free base)	PF-04691502
Brivanib (BMS-540215)	CP 673451	CCT137690
NVP-ADW742	AZD8055	CHIR-98014
Linsitinib (OSI-906)	PHT-427	AZ628

KU-55933	KRN 633	AMG458
GSK1904529A	AT7867	NVP-BGT226
PF-04217903	BMS 777607	PHA-848125
MLN8054	PD318088	Arry-380
Vatalanib dihydrochloride	KU-60019	ARQ 197 (Tivantinib)
U0126-EtOH	BS-181 HCl	ARRY334543
ZM-447439	BIRB 796 (Doramapimod)	Wortmannin
GDC-0879	Tie2 kinase inhibitor	NVP-BVU972
LY294002	TWS119	CH5424802
OSU-03012	BMS-265246	3-Methyladenine
Danusertib (PHA-739358)	AZD8330	Dinaciclib (SCH727965)
TAE684 (NVP-TAE684)	Neratinib (HKI-272)	Dovitinib Dilactic acid
BI 2536	KW 2449	MK-5108 (VX-689)
Foretinib	LY2784544	MK-2461
SGX-523	BGJ398 (NVP-BGJ398)	AZD2014
GSK690693	AST-1306	TAK-285
JNJ-38877605	AZD8931	INCB28060
PD 0332991 (Palbociclib) HCl	GSK461364	Tofacitinib
Triciribine	R406	Sotrastaurin (AEB071)
XL147	Raf265 derivative	WP1066
XL-184 free base	BMS 794833	AZD4547
Everolimus (RAD001)	NVP-BHG712	CEP33779
MLN8237 (Alisertib)	OSI-420 (Desmethyl Erlotinib)	Dabrafenib (GSK2118436)
AT9283	R935788	GDC-0068
Brivanib alaninate	PIK-293	INK 128
AG-490	AZ 960	BYL719
SNS-032 (BMS-387032)	Mubritinib (TAK 165)	Tyrphostin AG 879 (AG 879)
Barasertib (AZD1152-HQPA)	PP242	Torin 2
PLX-4720	Cyt387	NVP-TAE226
Roscovitine	SB590885	Tideglusib
SNS-314	Apatinib (YN968D1)	TPCA-1
E7080 (Lenvatinib)	CAL-101 (GS-1101)	Desmethyl Erlotinib (CP-473420)
CP-724714	PIK-294	Torin 1
TGX-221	Telatinib (BAY 57-9352)	SAR131675
WZ3146	BI6727 (Volasertib)	Semaxanib (SU5416)
CYC116	Palomid 529	Baricitinib (LY3009104)
WZ4002	WP1130	Golvatinib (E7050)
PD98059	BKM120 (NVP-BKM120)	IMD 0354
Regorafenib (BAY 73-4506)	cx-4945 (Silmitasertib)	WHI-P154
WZ8040	Indirubin	TG 100713
ENMD-2076	Quercetin (Sophoretin)	Piceatannol
PIK-90	Imatinib (Gleevec)	Tofacitinib citrate
PIK-75	Phenformin hydrochloride	VX-702
